# DNA methylation in repeat negative prostate biopsies as a marker of missed prostate cancer

**DOI:** 10.1186/s13148-019-0746-6

**Published:** 2019-10-30

**Authors:** Valentina Fiano, Daniela Zugna, Chiara Grasso, Morena Trevisan, Luisa Delsedime, Luca Molinaro, Paola Cassoni, Mauro Papotti, Franco Merletti, Olof Akre, Andreas Pettersson, Laura De Marco, Lorenzo Richiardi

**Affiliations:** 1 Cancer Epidemiology Unit-CeRMS, Department of Medical Sciences, University of Turin and CPO Piemonte, Via Santena 7, 10126 Turin, Italy; 2 Pathology Unit, A.O.U. Città della Salute e della Scienza Hospital, Turin, Italy; 3 Pathology Unit, Department of Medical Sciences, University of Turin, Turin, Italy; 4 Pathology Unit, Department of Oncology, University of Turin, Turin, Italy; 5 Cancer Epidemiology Unit, A.O.U. Città della Salute e della Scienza Hospital and CPO Piemonte, Turin, Italy; 6 Department of Molecular Medicine and Surgery, Karolinska Institutet and Department of Urology, Karolinska University Hospital, SE-17176 Stockholm, Sweden; 7 Clinical Epidemiology Unit, Department of Medicine, Solna, Karolinska Institutet, Stockholm, Sweden

**Keywords:** DNA methylation, Negative prostate biopsies, Prostate cancer, Prostate cancer diagnosis

## Abstract

**Background:**

Men often undergo repeat prostate biopsies because of suspicion of missed cancer. We assessed if (i) methylation of selected genes in prostate tissue vary with aging and (ii) methylation alterations in repeat biopsies predict missed prostate cancer.

**Methods:**

We conducted a case-control study among men who underwent at least two negative prostate biopsies followed by a sampling either positive (cases *n* = 111) or negative (controls *n* = 129) for prostate cancer between 1995 and 2014 at the University Hospital (Turin, Italy). Two pathology wards were included for replication purposes. We analyzed methylation of *GSTP1*, *APC*, *PITX2*, *C1orf114*, *GABRE*, and *LINE-1* in the first two negative biopsies. Conditional logistic regression was used to estimate odds ratios (ORs) and 95% confidence intervals (CIs) of the association between genes methylation and prostate cancer.

**Results:**

Age at biopsy and time interval between the two negative biopsies were not associated with methylation levels of the selected genes in neither cases nor controls. *GSTP1* methylation in the first and in the second negative biopsy was associated with prostate cancer detection [OR per 1% increase: 1.14 (95% CI 1.01–1.29) for the second biopsy and 1.21 (95% CI 1.07–1.37) for the highest methylation level (first or second biopsy)]. A threshold > 10% for *GSTP1* methylation corresponded to a specificity of 0.98 (positive likelihood ratio 7.87). No clear association was found for the other genes. Results were consistent between wards.

**Conclusions:**

Our results suggest that *GSTP1* methylation in negative prostate biopsies is stable over time and can predict missed cancer with high specificity.

## Background

Men with elevated prostate-specific antigen (PSA) levels and/or a digital rectal examination suspicious of cancer usually undergo standard prostate biopsy to confirm the presence of cancer [1, 2]. Prostate biopsies are affected by sampling error and have a false negative rate from 10% up to 30% [3–6]. As a consequence, many patients with a negative biopsy undergo one or several repeat biopsies, which are associated with pain, bleeding, and chance of serious infections from 0 to 6.3% [7, 8]. Although multiparametric magnetic resonance imaging (mp-MRI)-guided biopsies reduce the problem of false negative biopsies, the suspicion of cancer can remain high in a man with a previous negative round of biopsies [2].

Hence, there is a clinical need to identify diagnostic markers in morphologically benign tissue that can reduce the rate of repeat biopsies and missed cancer.

Several studies have shown that DNA hypermethylation of selected genes is present in non-tumor prostate tissue of men with prostate cancer, suggesting a diagnostic potential of aberrant DNA methylation in non-tumor tissue [9–15]. The biology of these alterations, if they change over time, and their potential clinical value are however poorly understood.

We studied men who underwent repeat prostate biopsies to understand (i) whether methylation alterations of selected genes in negative prostate tissue change over time and with aging or depend only on the specific characteristics of the prostate tissue in the sampled location and (ii) whether information on methylation alterations in repeat negative biopsies can be combined to predict the probability of a missed prostate cancer. We analyzed methylation of two genes [*GSTP1* (glutathione S-transferase P1) and *APC* (adenomatous polyposis coli)] suggested as potential diagnostic markers for prostate cancer [12–15], of three genes [*C1orf114* (chromosome 1 open reading frame 114), *GABRE* (gamma-aminobutyric acid receptor subunit epsilon), *PITX2* (paired-like homeodomain transcription factor 2)] previously associated with prostate cancer prognosis [16–18], and of *LINE-1* (long interspersed element-1), a marker of global methylation and a potential diagnostic and prognostic marker for prostate cancer [15–19].

## Results

The study population of this case-control study involved two wards and was nested among 18,402 patients who underwent at least one prostate sampling [i.e., biopsy, transurethral resection of prostate (TURP), or partial prostatectomy] between 1995 and 2014 at the Italian University Hospital “Città della Salute e della Scienza di Torino”, Torino, Italy. Cases and controls were defined as patients who underwent at least two biopsies negative for prostate cancer followed by a final sampling (i.e., the index sampling) that was either positive (cases) or negative (controls) for prostate cancer (Additional file 1: Figure S1). The study included 111 cases (86 Ward I and 25 Ward II) and 129 controls (100 Ward I and 29 Ward II); 34 subjects overlapped with a previous study [15]. Characteristics, including methylation levels, of cases and controls are reported in Table 1.

**Table 1:** Characteristics of cases and controls

Characteristic	Cases (*n* = 111)	Controls (*n* = 129)
Age at first biopsy, median years (IQR) (missing: 0)	66.2 (61.9–70.7)	65.6 (59.5–69.2)
Calendar year at first biopsy, median (IQR) (missing: 0)	2002 (98–04)	2003 (01–05)
Time interval between the first and second biopsy, median months (IQR) (missing: 0)	21.9 (11.9–37.1)	22.5 (12.7–41.5)
Time interval between the first biopsy and the index sampling, median months (IQR) (missing: 0)	44.5 (27.2–74.3)	45.9 (27.1–73.5)
Number of patients per ward (%) (missing: 0)		
Ward I	86 (77.5)	100 (77.5)
Ward II	25 (22.5)	29 (22.5)
*GSTP1* methylation %, median (IQR)		
First biopsy (missing: 8 cases, 14 controls)	3.25 (1.75–5.50)	2.75 (1.37–4.25)
Second biopsy (missing: 7 cases, 8 controls)	3.25 (2.00–6.00)	2.75 (1.75–3.75)
Difference between the second and the first biopsy (missing: 14 cases, 20 controls)	0.00 (− 2.25–2.25)	0.00 (− 1.00–1.25)
*PITX2* methylation %, median (IQR)		
First biopsy (missing: 18 cases, 23 controls)	8.75 (4.75–11.75)	8.25 (5.12–11.19)
Second biopsy (missing: 17 cases, 14 controls)	8.25 (5.50–12.19)	9.00 (5.75–12.87)
Difference between the second and the first biopsy (missing: 30 cases, 30 controls)	− 1.00 (−6.00–5.25)	0.00 (− 3.62–4.25)
*APC* methylation %, median (IQR)		
First biopsy (missing: 15 cases, 21 controls)	1.33 (1.00–3.08)	1.33 (1.33,3.33)
Second biopsy (missing: 16 cases, 17 controls)	1.33 (1.00–3.92)	1.33 (1.00–3.67)
Difference between the second and the first biopsy (missing: 29 cases, 33 controls)	0.00 (−1.33–1.83)	0.17 (− 0.33–1.42)
*C1orf114* methylation %, median (IQR)		
First biopsy (missing: 18 cases, 19 controls)	4.00 (2.00–8.50)	2.50 (1.50–5.50)
Second biopsy (missing: 12 cases, 9 controls)	5.00 (1.50–9.25)	4.50 (1.50–7.12)
Difference between the second and the first biopsy (missing: 28 cases, 23 controls)	0.00 (− 3.25–4.50)	0.50 (− 2.00–4.50)
*GABRE* methylation %, median (IQR)		
First biopsy (missing: 27 cases, 38 controls)	2.70 (1.00–6.30)	2.00 (1.00–4.90)
Second biopsy (missing: 28 cases, 25 controls)	2.60 (1.00–5.50)	1.90 (1.00–5.20)
Difference between the second and the first biopsy (missing: 44 cases, 51 controls)	− 0.20 (−1.90–2.40)	0.00 (− 2.00–1.80)
*LINE-1* methylation %, median (IQR)		
First biopsy (missing: 5 cases, 7 controls)	69.7 (66.0–74.0)	69.3 (66.3–72.6)
Second biopsy (missing: 3 cases, 3 controls)	69.3 (66.2–72.1)	69.0 (66.1–73.0)
Difference between the second and the first biopsy (missing: 24 cases, 30 controls)	0.00 (− 3.33–4.00)	0.17 (− 4.00–4.08)
Gleason score (missing: 2 cases)		
6	28 (25.7)	–
3 + 4	53 (48.6)	–
4 + 3	13 (11.9)	–
8+	15 (13.8)	–
PSA at the second biopsy, median (IQR) (missing: 22 cases, 19 controls)	9.15 (6.02–13.00)	8.00 (6.00–10.16)

*IQR* interquartile range, *PSA* prostatic-specific antigen

Additional file 1: Figure S2 reports the pairwise correlations between methylation levels of each selected gene within the first negative biopsy of cases and controls. All correlations were positive both in cases and controls, with an average correlation of 0.22 in cases and 0.20 in controls. The correlations were in general lower for *LINE-1* than for the five selected genes, for which the highest estimates were observed for *APC* and *PITX2* among cases (*r* = 0.48, *p* value = < 0.001) and *APC* and *GSTP1* among controls (*r* = 0.39, *p* value = < 0.001). Similar results (not shown) were found when the analyses were carried out in the second biopsy. With the exception of the correlation between methylation levels in *PITX2* and *GSTP1* and *PITX2* and *APC* (*p* = 0.008 and *p* = 0.017 respectively), there was no evidence of difference between cases and controls in the within biopsy gene-specific correlation coefficients (all *p* values > 0.10).

As shown in Additional file 2: Table S1, there was no evidence of gene-specific pairwise correlation between the first and the second biopsy, which were not necessarily matched on the same prostate anatomical region, with the exception of *LINE-1*, for which we estimated an *r* of 0.34 (*p* < 0.0001) in cases and 0.28 (*p* = 0.001) in controls. A positive correlation was also observed for *GSTP1* among controls (*r* = 0.23, *p* = 0.02), but less so among cases (*r* = 0.12, *p* = 0.25).

### Methylation changes in association with age and time between biopsies in cases and controls

**Fig. 1:**
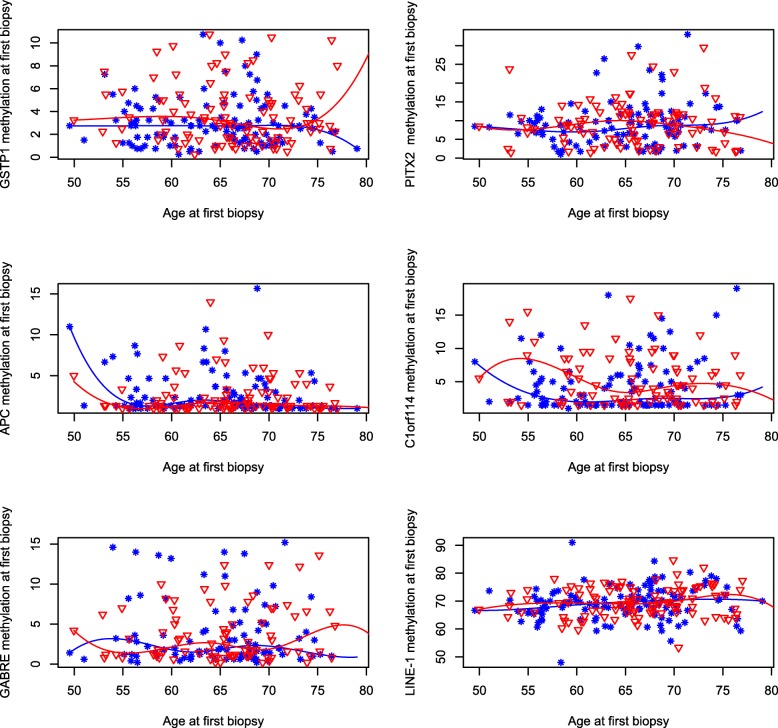
Gene-specific methylation levels at the first biopsy by age. Median methylation levels were modeled using restricted cubic splines with five knots and the fitted lines are presented graphically for cases (star, blue line) and controls (triangle, red line) separately

We evaluated the change in methylation levels with time in the prostate tissue of cases and controls using, first, a cross-sectional approach in which we assessed the association between age at the first biopsy and methylation levels of the selected genes, and second, a longitudinal approach in which we assessed the association between, on the one hand, the time between the first and the second biopsy and, on the other hand, the difference in methylation of each selected genes between the second and the first biopsy. The first, cross-sectional approach is potentially biased by patients’ heterogeneities with age at diagnosis, while the second, longitudinal approach is conducted within patients, and thus is not affected by their heterogeneities. Both in cases and controls, age at first biopsy was not associated with methylation levels in any of the selected genes; although there was variability in methylation levels among both cases and controls, the median levels remained constant with age (Fig. 1). The results were similar when we analyzed the association between age and methylation levels in the second biopsy (data not shown). The predicted values of gene-specific median methylation levels at the first biopsy at selected ages (55, 60, 65, 70, and 75 years) with 95% confidence intervals are reported in Additional file 2: Table S2.

**Fig. 2:**
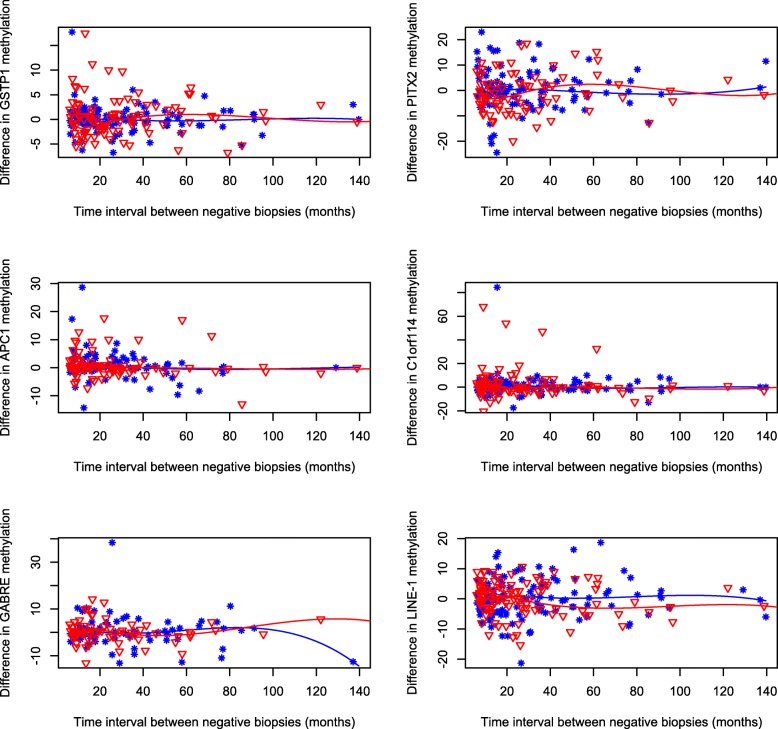
Differences in gene-specific methylation levels between the two negative biopsies by time interval. Median differences were modeled using restricted cubic splines with five knots and the fitted lines are presented graphically for cases (star, blue line) and controls (triangle, red line) separately

Consistently, analyses on the difference in methylation levels between the two biopsies of each selected gene by time interval revealed that, although there was a large variability in the differences of methylation levels between the first and second biopsies both among cases and controls, the median difference did not change over time for any of the genes (Fig. 2). The predicted values of the median differences in gene-specific methylation levels between the two biopsies, at selected time intervals (10, 20, 40, 60, 80, and 100 months), with 95% confidence intervals, are reported in Additional file 2: Table S3. The estimates were close to the null value and there was no evidence of a deviation from the null value over time.

### Methylation levels in selected genes and risk of prostate cancer

We estimated the association between methylation levels of the selected genes and the risk of prostate cancer detection (Table 2). The methylation level was treated as a continuous variable, and results were reported as the odds ratio (OR) of prostate cancer detection for a 1% increase in methylation. The main analyses focused on the methylation levels in the second biopsy in association with the risk of prostate cancer detection in the third sampling. We focused on the methylation levels in the second negative biopsy, instead of the first biopsy, as this study, by design, was constrained on having a second negative biopsy taken after the initial first negative biopsy (see the “Methods” section for further details). We also analyzed the highest methylation level (first or second biopsy) again in association with the risk of prostate cancer detection in the third sampling and for each of the selected genes. The methylation levels of *GSTP1* in the negative biopsies were associated with the risk of cancer diagnosis in the last sampling: the OR per 1% increase in methylation level was 1.14 (95% CI 1.01–1.29), and 1.21 (95% CI 1.07–1.37), for the highest methylation level (Table 2). We found no association between methylation levels of *PITX2*, *APC*, *GABRE*, or *LINE-1* and prostate cancer detection, but a weak association for *C1orf114* (Table 2). When all five genes and *LINE-1* were included in the same model, the association with *GSTP1* changed only marginally (data not shown). The adjustment for PSA did not substantially change the estimates.

**Table 2:** Association between gene-specific methylation (considered as a continuous variable, per each 1% increase) and the risk of prostate cancer detection

Gene	ORs of prostate cancer for methylation level in the second biopsy		ORs of prostate cancer for the highest^†^ methylation level between the first and the second biopsy	
	OR1 (95% CI)	OR2 (95% CI)	OR1 (95% CI)	OR2 (95% CI)
*GSTP1*	1.19 (1.06 to 1.33)	1.14 (1.01 to 1.29)	1.23 (1.10 to 1.39)	1.21 (1.07 to 1.37)
*PITX2*	0.98 (0.94 to 1.03)	0.99 (0.94 to 1.04)	1.02 (0.98 to 1.06)	1.02 (0.97 to 1.06)
*APC*	1.02 (0.95 to 1.09)	1.01 (0.94 to 1.09)	1.04 (0.97 to 1.10)	1.03 (0.96 to 1.10)
*C1orf114*	1.02 (0.99 to 1.06)	1.02 (0.98 to1.05)	1.04 (1.00 to 1.08)	1.03 (0.99 to 1.06)
*GABRE*	1.00 (0.94 to 1.06)	0.99 (0.93 to 1.06)	1.00 (0.95 to 1.06)	1.00 (0.94 to 1.06)
*LINE-1*	1.01 (0.95 to1.07)	0.99 (0.92 to 1.06)	1.00 (0.93 to 1.07)	0.97 (0.90 to 1.04)

*OR* odd ratio, *CI* confidence interval

OR1 adjusted for the matching variables (ward and time distance between the first biopsy and the index sampling)

OR2 adjusted for the matching variables, age and year at the first biopsy and prostatic-specific antigen (PSA) at the second biopsy (continuous variables are centered at their mean)

^†^for example: if methylation in *GSTP1* is 4% in the first biopsy and 7% in the second biopsy, the highest level used for this analysis is 7%

Limited to *GSTP1*, we also conducted analyses stratified by the Gleason score, to evaluate more aggressive (score of 4 + 3, or at least 8) and less aggressive (score of 6 or 3 + 4) prostate cancers, and by ward (Ward I and Ward II), for the purpose of validation. For *GSTP1*, the ORs for aggressive prostate cancer were similar or slightly higher compared to those for non-aggressive prostate cancer (Table 3). The association between *GSTP1* methylation and prostate cancer was present in both Wards, even if confidence intervals were wide in Ward II due to a smaller sample size.

**Table 3:** Association between *GSTP1* methylation (considered as a continuous variable, per each 1% increase) and the risk of prostate cancer detection stratified by Gleason score and ward

*GSTP1*	ORs of prostate cancer for methylation level in the second biopsy		ORs of prostate cancer for the highest^†^ methylation level between the first and second biopsy	
	OR1 (95% CI)	OR2 (95% CI)	OR1 (95% CI)	OR2 (95% CI)
Gleason score				
6 or 3 + 4	1.14 (1.02 to 1.27)	1.10 (0.97 to 1.25)	1.19 (1.06 to 1.35)	1.18 (1.03 to 1.34)
at least 4 + 3	1.33 (1.08 to 1.62)	1.22 (0.96 to 1.57)	1.27 (1.07 to 1.51)	1.21 (0.98 to 1.48)
ward				
Ward I (86 cases, 100 controls)	1.17 (1.03 to 1.32)	1.13 (0.99 to 1.30)	1.19 (1.06 to 1.34)	1.17 (1.03 to 1.33)
Ward II (25 cases, 29 controls)	1.26 (0.96 to 1.66)	1.08 (0.77 to 1.52)	1.61 (1.04 to 2.49)	1.58 (0.92 to 2.71)

*OR* odd ratio, *CI* confidence interval

OR1 adjusted for matching variables (ward and time distance between the first biopsy and the index sampling)

OR2 adjusted for the matching variables, age and year at the first biopsy and prostatic specific antigen (PSA) at the second biopsy (continuous variables are centered at their mean)

^†^For example, if methylation in *GSTP1* is 4% in the first biopsy and 7% in the second biopsy, the highest level used for this analysis is 7%

For GSTP1, a threshold > 10% revealed an OR of prostate cancer of 9.61 (95% CI: 1.07–86.3) for methylation level, and of 5.10 (95% CI: 1.33–19.6) for the highest methylation level. Table 4 reports the non-parametric values of specificity and sensitivity [and corresponding positive and negative likelihood ratios (LRs)] for different thresholds of *GSTP1* methylation. The LRs, i.e., LR+ when the test is positive and LR− when the test is negative, are based on sensitivity and specificity and give a straightforward summary measure of the informative value of a test, as the post-test probability can be easily calculated as a function of the pre-test probability and the LR (post-test odds = pre-test odds × LR).

**Table 4:** Non-parametric estimates of sensitivity, specificity, positive and negative likelihood ratios of prostate cancer detection in the third sampling, for increasing thresholds (from > 5 to > 10%) of *GSTP1* methylation observed in the first and second negative biopsy; 97 cases and 109 controls with measured *GSTP1* methylation in both the first and the second biopsy

Methylation threshold	*n* positive cases	*n* negative cases	*n* positive controls	*n* negative controls	Sens	Spec	LR+	LR−
	The highest methylation level between the first and second biopsy							
> 5%	44	53	22	87	0.45	0.80	2.25	0.68
> 6%	32	65	12	97	0.33	0.89	3.00	0.75
> 7%	27	70	8	101	0.28	0.93	3.79	0.78
> 8%	19	78	4	105	0.20	0.96	5.34	0.83
> 9%	17	80	3	106	0.17	0.97	6.37	0.85
> 10%	14	83	2	107	0.14	0.98	7.87	0.87

*Sens* sensitivity, *Spec* specificity, *LR* likelihood ratio

A threshold of > 10% corresponded to a specificity of 0.98 and a LR+ of 7.87, although sensitivity was low and LR− was close to 1.0.

## Discussion

We found, both in cases and controls, that neither the age at prostate biopsy nor the time interval between two negative biopsies were associated with methylation of *GSTP1*, *APC*, *C1orf114*, *GABRE*, *PITX2*, or *LINE-1* in non-tumor prostate tissue. This occurred even if there was a large variation in methylation levels both among patients and between two biopsies of the same patient, suggesting that methylation levels are specific to the specific sampling location in the prostate tissue, but do not follow specific changing patterns with time in histologically benign tissue. *GSTP1* methylation in the first and the second negative biopsy was associated with the risk of cancer detection in the final sampling, while no clear association was found for the other genes. These results suggest that *GSTP1* methylation in negative prostate biopsies is stable over time and that *GSTP1* methylation can predict a missed cancer. Given the large differences in *GSTPI* methylation levels between patients’ biopsies, these results suggest that the diagnostic value of *GSTP1* methylation can be further improved by analyzing *GSTP1* methylation levels in repeat biopsies.

A previous study by Kwabi-Addo and colleagues [20] on non-tumor prostate tissue, obtained from organ donors and patients who underwent cystoprostatectomy for bladder cancer, found that methylation of selected genes (including *GSTP1*) was positively associated with the patient’s age. This finding is only in apparent contradiction with our results, as there are possible explanations. First, they included a large age range, from 17 to 84 years; from a visual inspection of Fig. 3 reported in the Kwabi-Addo and colleagues article [20], the slope of the association between methylation and age was strongly affected by patients aged less than 40 years, who were not included in our study as men are rarely scrutinized for prostate cancer at that age. The stability over time that we observed in our study may thus occur only at older ages. Second, the association between *GSTP1* methylation and age was mainly due to seven cystoprostatectomy patients with much higher *GSTP1* methylation levels than those found in non-tumor prostate tissues matched to prostate cancer tissues of 12 patients included in the study. In our study, the lack of association between age at sampling and *GSTP1* methylation was supported by results from the longitudinal analyses carried out within patients, which are not affected by patients’ heterogeneity. Our results thus suggest that *GSTP1* hypermethylation is not due to aging but rather probably due to epigenetic deregulations occurring early in cancerogenesis.

The finding of an association between *GSTP1* hypermethylation in non-tumor prostate tissue from a negative biopsy and the risk of prostate cancer detection in a later biopsy was replicated in both wards included in our study. Our findings are also consistent with the results of previous studies on *GSTP1* methylation in non-tumor tissue as a potential marker for prostate cancer [9, 10, 13–15]. We found that it is possible to improve the prediction by combining information on *GSTP1* hypermethylation from multiple negative biopsies, even when obtained some years apart. It is thus likely that the predictive information from different biopsy procedures resembles the same information that could be obtained by the analysis of multiple cores of the same biopsy procedure. Finally, we found that a high threshold of methylation of *GSTP1* is associated with a very high specificity, suggesting that this test could identify missed prostate cancer with a minimal increase in false positives. *GSTP1* methylation testing in non-tumor tissue could thus potentially be combined with tests with high sensitivity, such as targeted biopsy after mp-MRI [2, 6, 21], to improve overall diagnostic accuracy.

Steward and colleagues [13] and others [9, 10] have suggested that the combination of methylation of *APC* and *GSTP1* could outperform the use of methylation of *GSTP1* alone. In our study, however, methylation of *APC* was not associated with prostate cancer detection. Interestingly, in the study by Steward and colleagues, *GSTP1* methylation was associated with a LR+ of 3.1 and a LR− of 0.67; when *GSTP1* was combined with *APC*, the LR− improved to 0.53, but the LR+ worsen to 1.7 (calculated by us on the basis of the sensitivity and specificity estimates reported in their Table 2). These findings would suggest that, if the aim is to maximize specificity, *GSTP1* testing alone could outperform the combination of *APC* and *GSTP1*. Depending on the possible clinical uses, it could be discussed on whether to maximize LR+ or LR− or both and whether adding *APC* methylation would actually improve the test or not. It should be also noted that in our study, we assessed different CpG sites and used a different technique to assess *APC* methylation than in the study by Stewart et al.

The fact that we did not find an association with prostate cancer detection for the other analyzed genes, with the possible exception of *C1orf114*, suggests that the association between *GSTP1* methylation and prostate cancer is not a consequence of a general alteration of the DNA methylome; nevertheless, we found a non-negligible positive correlation between genes in the same biopsy, which is consistent with the concept that the methylation pattern is a local characteristic of the prostate tissue. The lack of association for the other genes may also imply that the alteration in their methylation is a later event in cancerogenesis. It should be noted that these findings are consistent with previous studies that linked methylation of those genes with prostate cancer progression [16–18].

Although to our knowledge this is the first study that analyzed the change in methylation between negative biopsies and its relationship with a missed prostate cancer, our study has limitations. First, our study was restricted to patients who received at least three biopsies and these results are not necessarily generalizable to patients at their first biopsy. Second, the sample size was not sufficient to obtain strong evidence on the potential specificity of the *GSTP1* methylation for aggressive prostate cancer (as opposed to non-aggressive cancers). Third, we analyzed a limited number of CpGs for each selected gene; a larger number of CpGs could give a more precise estimate of the gene-specific methylation levels.

In conclusion, our data suggest that *GSTP1* methylation in negative prostate biopsies is stable over time and can predict missed cancer with high specificity.

## Methods

### Study population

We conducted a case-control study nested among 18,402 patients who underwent at least one prostate sampling [i.e., biopsy, transurethral resection of prostate (TURP) or partial prostatectomy] between 1995 and 2014 at the Italian University Hospital “Città della Salute e della Scienza di Torino”, Italy. For validation purposes, we involved both pathology wards at the hospital (hereafter identified as Ward I and Ward II). Out of the 18,402 patients, we identified 761 patients who underwent at least two samplings negative for prostate cancer followed by a final sampling (i.e., the index sampling) that was either positive (potential cases, *N* = 230) or negative for prostate cancer (non-cases, *N* = 531) (Additional file 1: Figure S1). We excluded patients whose negative samplings obtained before the index sampling were taken less than 6 months apart. We also excluded patients whose at least one of the negative sampling was a TURP or a partial prostatectomy as the prostate transition zone has a different methylation profile than the prostate peripheral zone (52 cases and 95 non-cases) [15–19]. Then, we sampled controls and matched cases to controls with a 1:1 ratio, on the pathology ward and the time interval between the first biopsy and the index sampling. After visual inspection of the slides and reading of the pathology reports, we excluded 22 cases and 16 controls for whom at least one of the two negative samplings was not a biopsy, 1 control whose biopsy was obtained only from the transitional zone, 6 cases and 3 controls with inadequate amount of tissue for molecular analyses, 2 cases with prostate cancer in one of the first two biopsies, 4 cases with bladder cancer that were erroneously included in the initial series of patients and 6 cases who had a diagnoses of atypical small acinar proliferation (ASAP) or high-grade prostatic intraepithelial neoplasia (HGPIN) and not prostate cancer. Incomplete strata with no cases or no controls were excluded, leading to the exclusion of 7 cases and 1 control; 111 cases and 129 controls remained in the study (Additional file 1: Figure S1).

Information on age at biopsy, tumor histology, and age value was obtained from the pathology reports. Since information on PSA was available for at least 80% of the subjects at the second biopsy, and only for 59% of the cases and 67% of the controls at the first biopsy, we only kept information at the second biopsy in the study. For all subjects, we collected blocks of formalin-fixed paraffin-embedded (FFPE) prostate tissue from the first two negative biopsies. If more than one FFPE block was available, we randomly selected one block, which implies that the repeat samples analyzed for the same patient were not necessarily obtained from the same anatomical region. For cases and controls with more than two negative biopsies before the index sampling, we selected the first and the last available biopsy to maximize the time distance between the two negative biopsies.

The diagnostic slides of the cases were reviewed by an uropathologist to assign a standardized contemporary Gleason score. For two cases, the diagnostic slides could not be evaluated and the Gleason score was considered missing.

### Molecular analysis

For each FFPE block of the two first negative biopsies, we cut 3 to 5 (10 μm thick) sequential sections and extracted DNA, avoiding areas of chronic inflammation and fibromuscular stroma, using the QIAamp® DNA FFPE Tissue Kit (Qiagen, Hilden, Germany). Genomic DNA underwent bisulphite modification using the EpiTect 96 Bisulfite Kit (Qiagen) and methylation was analyzed using PyroMark Q24 MDx (Qiagen). PCR primers that amplify the gene promoter region containing the target CpG sites, and the sequencing primers were designed with the software PyroMark Assay Design 2.0 (Qiagen) (Additional file 2: Table S4). We performed PCR reactions using PyroMark PCR kit (Qiagen) following the manufacturer’s instructions, except for the annealing temperature (Additional file 2: Table S4). Methylated and unmethylated controls (EpiTect Control DNA, methylated and EpiTect Control DNA, unmethylated, Qiagen) were included in each PCR and pyrosequencing run. For each gene, we calculated the mean methylation levels among the target CpG sites (3 for *APC* and *LINE-1*, 4 for *GSTP1* and *PITX2* and 5 for *GABRE*, 2 for *C1orf114*) as in general they were strongly correlated within the same gene.

For each matched case-control stratum, all DNA samples were analyzed in the same batch and randomly allocated within the plate. To preserve the matching within batches, we did not re-run analyses in case of failure, which ranged from 25 to 30% (*GABRE*) to approximately 5% (*GSTP1* and *LINE-1*).

### Statistical analysis

We used the Spearman rank coefficient to estimate the pairwise correlation of methylation levels between genes in the same biopsy or between two patients’ biopsies in the same gene. We applied the standard Fisher’s *z*-transformation to the Spearman coefficients to compare between cases and controls the correlation estimates [22]. We then used the Benjamini-Yekuteli method to control for multiple comparisons [23].

Analyses of the change in methylation levels with time/aging were carried out in cases and controls separately. For each of the selected genes, we analyzed the association between (i) methylation levels and age at prostate biopsy; (ii) the difference in methylation levels between the first and the second biopsy and the time interval between the two biopsies. Both analyses provide information on whether time/aging is associated with increasing or decreasing methylation; the former through a cross-sectional observation of methylation levels in men aged 50 to 80 years (i.e., the age distribution among study subjects), and the latter through a within-subject longitudinal observation of changes in methylation levels on a time interval from 6 to 140 months (i.e., the minimum and maximum time interval between the first and the second negative biopsy). Because the distribution of the methylation levels did not meet the normality assumption, we used quantile regression to model the medians of the methylation levels and the difference in methylation levels between the first and the second sample [24]. Age and time were modeled using restricted cubic splines with five knots. Because of the difficulty in interpreting the coefficients for a variable modeled using splines, we calculated the predicted values (with 95% confidence intervals) of gene-specific median methylation levels at the first biopsy at selected ages (55, 60, 65, 70, and 75 years) (Additional file 2: Table S2). Similarly, we calculated the predicted values of the median differences in gene-specific methylation levels between the two biopsies at selected time intervals (10, 20, 40, 60, 80, and 100 months) (Additional file 2: Table S3).

We imputed missing values in gene methylation using multiple imputation by chained equations (MICE), assuming that the data were missing at random (MAR) [25]. The imputation model included demographic and clinical characteristics, including PSA levels at the second biopsy, as well as the methylation levels of the selected genes. Gleason score was not imputed but it was used as a predictor in the imputation models. We created 20 imputed datasets and combined their estimates according to Rubin’s rule.

For each imputed dataset, we used conditional logistic regression to estimate odds ratio (ORs) and corresponding 95% confidence intervals (CIs) of the association between methylation levels and prostate cancer detection. Each of the genes was introduced separately in the model. All models were inherently adjusted for the matching variables (time distance between the two biopsies and ward) and batch. We further adjusted for age, calendar year at the first biopsy (both introduced as continuous and centered at their mean), and PSA levels at the second biopsy.

We investigated the association between methylation levels in the second biopsy and prostate cancer detection and, separately, the association between the highest methylation level (first or second biopsy) and prostate cancer detection. We did not investigate the association between methylation levels in the first biopsy and prostate cancer detection because the estimates would have been biased by the fact that, by design, we conditioned on the second sampling being negative for cancer.

Methylation was modeled as a continuous variable. For *GSTP1*, we also used a threshold of > 10%, which was higher than the threshold of ≥ 5% that we used in a previous study [15], in order to enhance specificity. On the non-imputed data, we calculated non-parametric estimates of sensitivity, specificity, and positive and negative likelihood ratios (LRs) for levels of methylation from > 5 to > 10%.

The analyses described above were conducted on the whole study sample, separately by Ward I and Ward II for the purpose of validation, and separately for “aggressive” (Gleason score of at least 4 + 3) and “non-aggressive” (Gleason score < 4 + 3) prostate cancer.

## Supplementary information

**Additional file 1: Figure S1.** Flow-chart diagram showing the case and control selection. Figure S2. Spearman rank correlation coefficients between gene-specific methylation levels within biopsy in cases and controls.

**Additional file 2: Table S1.** Spearman correlation coefficients of gene-specific methylation levels between the two biopsies in cases and controls. **Table S2.** Predicted values of gene-specific median methylation levels at the first biopsy at selected ages, using quantile regression with age modelled by restricted cubic splines. Table S3. Predicted values of the median differences in gene-specific methylation levels between the two biopsies, at selected time intervals, using quantile regression with time modelled by restricted cubic splines. Table S4. Gene IDs, primer sequences, pyrosequencing assays and PCR annealing temperatures and number of CpG analysed per gene.

## Data Availability

The datasets generated during and/or analyzed during the current study are available from the corresponding author on reasonable request of qualified researchers for the purpose of academic, non-commercial research.

## Abbreviations

*APC*: Adenomatous polyposis coli
ASAP: Atypical small acinar proliferation
*C1orf114*: Chromosome 1 open reading frame 114
CIs: Confidence intervals
FFPE: Formalin fixed paraffin embedded
*GABRE*: Gamma-aminobutyric acid receptor subunit epsilon
*GSTP1*: Glutathione S-transferase P1
HGPIN: High-grade prostatic intraepithelial neoplasia
*LINE-1*: Long interspersed element-1
LRs: Likelihood ratios
MAR: Missing at random
MICE: Multiple imputation by chained equations
mp-MRI: Multiparametric magnetic resonance imaging
ORs: Odds ratio
*PITX2*: Paired-like homeodomain transcription factor 2
PSA: Prostate-specific antigen
TURP: Transurethral resection of prostate
