# Supplementary material for: DNA methylation in repeat negative prostate biopsies as a marker of missed prostate cancer
Source: Clin Epigenetics. 2019 Oct 30;11:152. doi: 10.1186/s13148-019-0746-6 (PMC6820908; doi:10.1186/s13148-019-0746-6)
Supplement: Supplementary file 1 — Additional file 1: Figure S1. Flow-chart diagram showing the case and control selection. Figure S2. Spearman rank correlation coefficients between gene-specific methylation levels within biopsy in cases and controls. [file 13148_2019_746_MOESM1_ESM.doc]

**Additional file 1**

**Figure S1** Flow-chart diagram showing the case and control selection

**
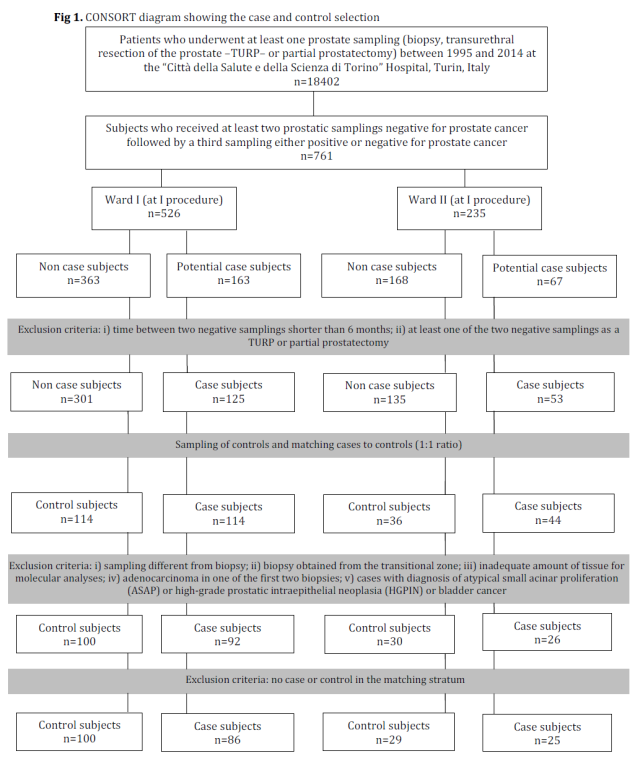
**

**Figure S2** Spearman rank correlation coefficients between gene-specific methylation levels within biopsy in cases and controls
