# Supplementary material for: DNA methylation in repeat negative prostate biopsies as a marker of missed prostate cancer
Source: Clin Epigenetics. 2019 Oct 30;11:152. doi: 10.1186/s13148-019-0746-6 (PMC6820908; doi:10.1186/s13148-019-0746-6)
Supplement: Supplementary file 2 — Additional file 2: Table S1. Spearman correlation coefficients of gene-specific methylation levels between the two biopsies in cases and controls. Table S2. Predicted values of gene-specific median methylation levels at the first biopsy at selected ages, using quantile regression with age modelled by restricted cubic splines. Table S3. Predicted values of the median differences in gene-specific methylation levels between the two biopsies, at selected time intervals, using quantile regression with time modelled by restricted cubic splines. Table S4. Gene IDs, primer sequences, pyrosequencing assays and PCR annealing temperatures and number of CpG analysed per gene. [file 13148_2019_746_MOESM2_ESM.doc]

**Additional file 2**

**Table S1** Spearman correlation coefficients of gene-specific methylation levels between the two biopsies in cases and controls

| Gene | Cases | | Controls | |
| --- | --- | --- | --- | --- |
|  | Spearman correlation | p-value | Spearman correlation | p-value |
| *GSTP1* | 0.12 | 0.25 | 0.23 | 0.02 |
| *APC* | -0.06 | 0.59 | 0.16 | 0.12 |
| *PITX2* | 0.00 | 1.00 | -0.09 | 0.39 |
| *C1orf14* | 0.01 | 0.95 | -0.04 | 0.67 |
| *GABRE* | 0.13 | 0.29 | 0.19 | 0.09 |
| *LINE-1* | 0.34 | <0.001 | 0.28 | 0.001 |

**Table S2** Predicted values of gene-specific median methylation levels at the first biopsy at selected ages, using quantile regression with age modelled by restricted cubic splines

|  | Estimated median methylation levels (95% CI) | | | | |
| --- | --- | --- | --- | --- | --- |
|  | 55 years | 60 years | 65 years | 70 years | 75 years |
| Cases |  |  |  |  |  |
| *GSTP1* | 3.47  (0.94;6.01) | 3.57  (1.14;5.99) | 3.18  (1.49;4.87) | 2.51  (0.84;4.18) | 3.33  (1.42;5.23) |
| *PITX2* | 7.63  (5.80;9.45) | 9.10  (7.25;10.95) | 9.99  (7.23;12.76) | 8.86  (6.18;11.54) | 7.07  (1.82;12.31) |
| *APC* | 1.15  (0.34;1.96) | 1.23  (0.62;1.84) | 1.81  (1.05;2.57) | 1.49  (0.83;2.15) | 1.32  (0.95;1.69) |
| *GABRE* | 1.38  (-3.23;6.00) | 2.14  (-0.22;4.49) | 2.53  (1.08;3.96) | 1.86  (-0.87;4.59) | 4.16  (-2.31;10.62) |
| *C1ORF114* | 8.41  (3.52;13.29) | 5.64  (2.52;8.77) | 3.39  (0.84;5.94) | 4.40  (2.26;6.55) | 4.56  (1.88;7.24) |
| *LINE-1* | 69.07  (63.08;75.05) | 69.28  (63.30;75.27) | 69.00  (65.57;72.43) | 69.90  (66.86;72.93) | 72.30  (66.45;78.16) |
| Controls |  |  |  |  |  |
| *GSTP1* | 2.75  (1.34;4.16) | 2.76  (2.08;3.43) | 2.73  (2.05;3.42) | 2.79  (1.78;3.79) | 2.40  (0.94;3.87) |
| *PITX2* | 7.49  (5.48;9.50) | 6.97  (4.61;9.33) | 7.89  (5.76;10.01) | 8.73  (6.25;11.22) | 9.32  (5.36;13.29) |
| *APC* | 1.96  (0.33;3.59) | 1.59  (0.99;2.20) | 1.93  (1.37;2.50) | 1.36  (0.78;1.93) | 1.04  (0.57;1.51) |
| *GABRE* | 3.09  (1.50;4.68) | 1.79  (0.35;3.22) | 1.84  (0.38;3.30) | 2.24  (0.17;4.32) | 1.25  (-2.32;4.82) |
| *C1ORF114* | 3.30  (0.84;5.75) | 2.00  (0.90;3.09) | 2.25  (0.90;3.61) | 2.50  (1.02;3.97) | 2.65  (-0.14;5.44) |
| *LINE-1* | 67.29  (64.87;69.70) | 68.66  (67.27;70.05) | 69.72  (68.00;71.43) | 70.38  (67.87;72.90) | 70.65  (65.76;75.55) |

CI confidence interval

**Table S3** Predicted values of the median differences in gene-specific methylation levels between the two biopsies, at selected time intervals, using quantile regression with time modelled by restricted cubic splines

|  | Estimated median difference in the methylation levels (95% CI) | | | | | |
| --- | --- | --- | --- | --- | --- | --- |
|  | 10 months | 20 months | 40 months | 60 months | 80 months | 100 months |
| Cases |  |  |  |  |  |  |
| *GSTP1* | -1.66  (-5.28;1.96) | -0.95  (-5.07;3.18) | 0.54  (-6.00;7.08) | 0.99  (-0.90;2.87) | 0.75  (-13.05;14.56) | 0.19  (-15.40;15.78) |
| *PITX2* | -2.50  (-9.27;4.28) | -2.61  (-11.83;6.62) | 1.31  (-11.0.8;13.70) | 2.39  (-3.03;7.81) | 1.40  (-20.36;23.17) | -0.41  (-35.00;34.17) |
| *APC* | 0.31  (-1.40;2.03) | 0.02  (-1.82;1.87) | -0.02  (-3.20;3.15) | -0.15  (-1.57;1.26) | -0.34  (-5.16;4.47) | -0.51  (-8.19;7.17) |
| *GABRE* | -0.80  (-6.38;4.78) | 0.78  (-10.96;12.53) | -1.25  (-19.98;17.49) | -0.95  (-6.26;4.36) | 1.11  (-40.03;42.25) | 3.67  (-34.64;41.99) |
| *C1ORF114* | 0.23  (-4.76;5.23) | -0.75  (-7.95;6.45) | -0.03  (-9.72;9.67) | -0.14  (-4.52;4.23) | -0.81  (-15.55;13.92) | -1.48  (-25.29;22.32) |
| *LINE-1* | 0.07  (-5.90;6.05) | 0.48  (-7.78;8.75) | -1.98  (-22.03;18.06) | -3.05  (-10.25;4.14) | -2.93  (-47.04;41.17) | -2.32  (-49.90;45.27) |
| Controls |  |  |  |  |  |  |
| *GSTP1* | -0.01  (-0.77;0.74) | 0.02  (-0.64;0.69) | -0.22  (-1.02;0.58) | -0.31  (-1.37;0.74) | -0.14  (-2.09;1.81) | 0.10  (-2.90;3.10) |
| *PITX2* | -0.37  (-3.36;2.62) | -0.013  (-2.22;2.19) | 0.08  (-3.42;3.57) | -0.78  (-4.67;3.11) | -1.39  (-5.05;2.28) | -1.43  (-7.57;4.71) |
| *APC* | -0.11  (-6.42;6.20) | 0.41  (-8.80;9.61) | -0.07  (-9.84;9.70) | -0.50  (-67.5;66.55) | -0.58  (-202.1;200.9) | -0.44  (-1341.2;1340.3) |
| *GABRE* | 0.11  (-5.33;5.56) | -0.02  (-9.93;9.89) | -0.30  (-17.95;17.34) | 0.88  (-22.65;24.42) | 1.96  (-3.42;7.35) | 1.02  (-248.10;250.13) |
| *C1ORF114* | 1.21  (-1.51;3.93) | 1.99  (0.26;3.72) | 0.12  (-1.73;1.97) | -0.84  (-3.43;1.74) | -0.78  (-5.46;3.90) | -0.24  (-7.14;6.65) |
| *LINE-1* | -0.67  (-3.14;1.79) | 0.80  (-2.94;4.54) | 0.34  (-2.45;3.14) | 0.26  (-2.60;3.12) | 0.73  (-2.72;4.19) | 1.14  (-4.60;6.88) |

CI confidence interval
